# Choroidal Osteoma With Choroidal Excavation Due to Decalcification Five Years After Photodynamic Therapy: A Case Report

**DOI:** 10.7759/cureus.59581

**Published:** 2024-05-03

**Authors:** Seiya Inamoto, Hisashi Matsubara, Erikio Uchiyama, Kumiko Kato, Mineo Kondo

**Affiliations:** 1 Ophthalmology, Mie University School of Medicine, Tsu, JPN

**Keywords:** choroidal excavation, decalcification, pdt, photodynamic therapy, choroidal osteoma

## Abstract

A choroidal osteoma (CO) is a relatively rare, benign tumor with ossification that develops in the choroid and undergoes enlargement and decalcification in its natural course. Photodynamic therapy (PDT) is used to induce decalcification, but there are few reports on individual cases treated with PDT. A 47-year-old Japanese man who had reduced decimal visual acuity (VA) of the right eye to 0.7 due to a CO away from the fovea was treated with PDT. The PDT resulted in a partial decalcification of CO, and the visual acuity improved to 1.0. However, the tumor slowly expanded, eventually reaching the central fovea. Decalcification and focal choroidal excavation occurred during this natural course of the disease. Although his metamorphopsia worsened, his VA was maintained at 1.0. This case highlights that a CO partially decalcified after PDT can still enlarge and decalcify over several years. These findings indicate the need for careful and continuous monitoring of eyes with a CO.

## Introduction

A choroidal osteoma (CO) is a relatively rare, benign, ossifying tumor originating in the choroid. It was first described in 1978 [1-3] and is more common in young women in their 20s and 30s. It is often unilateral, and the vision is preserved in the early stages of CO. However, COs are progressive and a prolonged disease progression has been associated with a reduction in vision [4,5]. Subretinal fluid (SRF) without choroidal neovascularization (CNV) and CNV have been reported to be the cause of vision reduction [6,7]. The location of the CO affects the visual prognosis. Tumors away from the central fovea often have preserved vision, whereas tumors extending into the central fovea have a poor visual prognosis. In its natural course, a CO may expand and contract with decalcification [7,8]. In eyes with a CO, there is a displacement of the outer retina and choroid outward due to tissue loss. This then results in a focal choroidal excavation (FCE) [9]. If the tumor expands into the central fovea, decalcification leads to retinal pigment epithelium (RPE) and photoreceptor degeneration resulting in visual impairment.

Intravitreal injections of anti-vascular endothelial growth factor agents and photodynamic therapy (PDT) have been reported to help maintain good visual acuity [6,10]. PDT for COs away from the central fovea has successfully led to decalcification and slowing of the progression of the tumor with a decalcification in PDT-treated areas [11]. In an earlier study that examined cases of spontaneous decalcification, COs that had decalcified at the time of the initial diagnosis did not have a further tumor expansion [7]. However, there are several reports on the course of cases with decalcification after PDT.

We previously reported a case in which SRF disappeared after PDT with excellent results [12]. Now, we present new findings on the long-term follow-up. Thus, the purpose of this study was to present our findings in a case of CO in which an extrafoveal CO with SRF in the central fovea was treated with PDT and partially decalcified. Still, the tumor expanded and reached the central fovea, and then spontaneously decalcified and formed an FCE.

This article was previously presented as a meeting abstract at the 2023 Japanese Retina and Vitreous Society (JRVS) Annual Meeting on November 24, 2023.

## Case presentation

**Figure 1:**
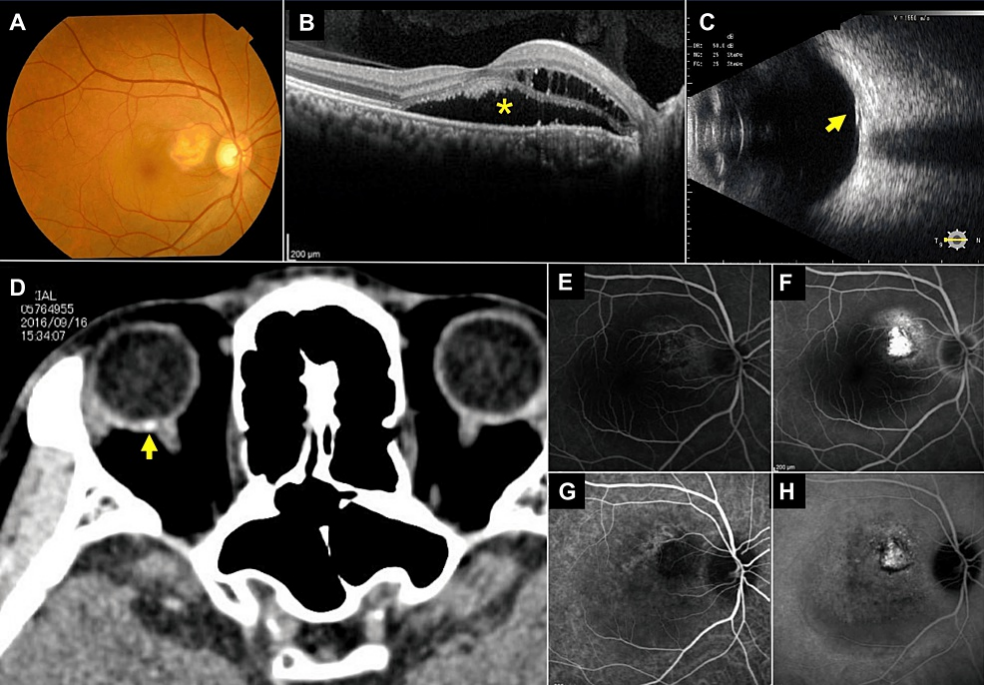
Multimodal images of a choroidal osteoma (CO) at the initial visit. A: Fundus photograph shows a yellowish-white and orange lesion between the optic disc and fovea. B: Optical coherence tomography (OCT) image shows subretinal fluid (SRF) (asterisk) and retinal edema in the horizontal cross-sectional image of the central fovea. C: Ultrasound image shows a hyperreflective lesion with acoustic shadowing at the posterior pole (arrow). D: Computed tomography image of the right orbital region showing a highly absorptive area on the posterior wall of the right eye (arrow). E: Fluorescein angiogram (FA) in the early phase shows hyperfluorescence with mottled hypofluorescence of the CO. F: FA image in the late phase shows hyperfluorescence of the tumor. G: Indocyanine angiogram (ICGA) in the late phase shows hypofluorescence for tumor lesions. H: ICGA in the late phase shows hyperfluorescence of the CO.

A 47-year-old Japanese male presented to our clinic complaining of visual field distortions and vision loss in his right eye. Ophthalmologic examination found that his decimal best-corrected visual acuity (BCVA) was 0.7 in the right eye and 1.2 in the left eye. Slit-lamp examination detected no abnormalities in the anterior chamber, lens, and vitreous cavity. Fundus examination revealed a yellowish-white to orangish-red elevated lesion near the macula and superior to the optic disc in the right eye (Figure 1A). Optical coherence tomography (OCT) revealed SRF in the macular region of the right eye (Figure 1B). B-mode ultrasound imaging identified a hyperechoic lesion with an acoustic shadow at the posterior pole (Figure 1C). Orbital computed tomography scans disclosed a hyperdense area in the posterior wall of the right eye (Figure 1D). Early-phase fluorescein angiography showed leakage with mottled hypofluorescence in the area of the raised lesion (Figure 1E). There was also fluorescein leakage and tissue staining in the late phase (Figure 1F). Indocyanine green angiography showed a filling delay in the early phase (Figure 1G) and leakage in the late phase (Figure 1H). These findings led to a diagnosis of CO in the right eye. The fundus of the left eye was completely normal.

**Figure 2:**
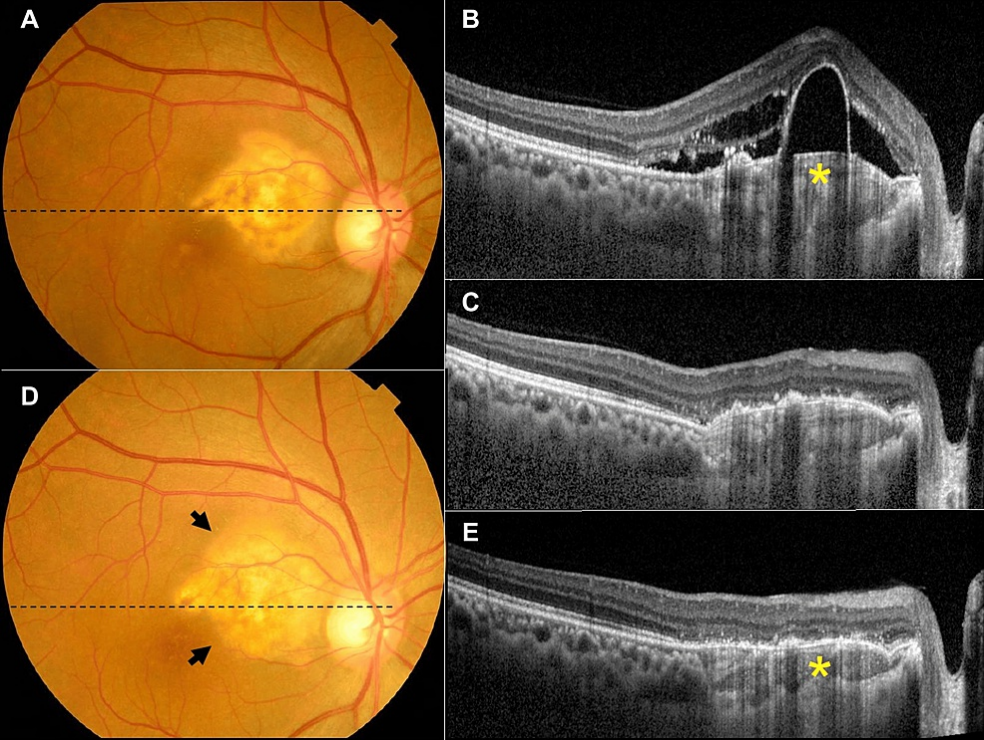
Fundus photographs and horizontal optical coherence tomography (OCT) images before and after the full-dose photodynamic therapy (PDT). A and B: Fundus photograph before the full-dose PDT shows the choroidal osteoma (CO) is larger than that at the initial visit, and the OCT image shows an elevation of the retinal pigmental epithelium due to the CO (asterisk), subretinal fluid (SRF), pigment epithelium detachment (PED), and retinal edema. C: OCT image one month after the full-dose PDT shows the absence of SRF, PED, and retinal edema. D and E: Six months after the full-dose PDT, the fundus photograph shows a partially enlarged CO lesion (arrows), and the OCT images show a decrease in the elevation of the RPE due to the decalcification of the CO (asterisk).

**Figure 3:**
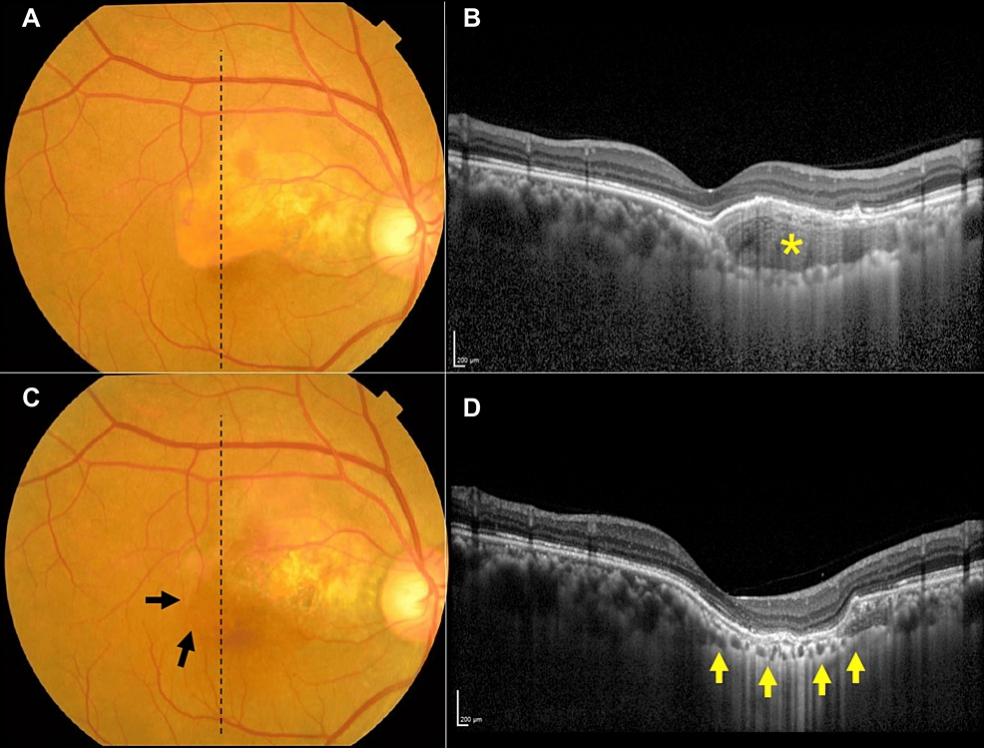
Fundus photographs and vertical optical coherence tomography (OCT) images before and after the decalcification and formation of choroidal excavation. A: Five years and three months after the full-dose photodynamic therapy (PDT). The choroidal osteoma (CO) has expanded to the subfoveal area. B: OCT image shows an elevation of the retinal pigmental epithelium (RPE) due to the CO lesion (asterisk). The RPE line at the central fovea is continuous. C: Five years and nine months after the full-dose PDT, the CO lesion is decalcified, and the orange lesion is not present. (arrows). D: OCT image shows the deeply depressed RPE and the wide choroidal excavation (arrows). The RPE line at the central fovea is intact, but part of the RPE line above the central fovea is discontinuous.

Four intravitreal injections of bevacizumab (1.25 mg/0.05 mL) (IVB) were administered every month to try to resolve the SRF, but the IVB failed. Next, PDT with a half dose of verteporfin was performed six months after the initial diagnosis. The SRF did not disappear, and an expansion was observed (Figures 2A, 2B). Four months later, PDT with full-dose verteporfin combined with IVB was performed, and the SRF disappeared 1.5 months later (Figure 2C). The decimal BCVA improved to 1.2. Six months later, the tumor had partially decalcified, and the bumps caused by the tumor were reduced (Figures 2D, 2E). There was no recurrence of the SRF. Two years and five months after the full-dose PDT, IVB was performed because of CNV with hemorrhage away from the central fovea. The hemorrhage was then absorbed, and the CNV subsided, but two months later, a new hemorrhage from the CNV appeared. Subsequently, another IVB was performed. Four years and four months after the last full-dose PDT, CNV with hemorrhage and pigmental epithelium detachment (PED) developed without SRF, and then the hemorrhage resolved spontaneously. Five years and two months after the full-dose PDT, the tumor expanded into the subfoveal area accompanied by irregularities in the RPE over the tumor (Figures 3A, 3B). Five years and 11 months after the full-dose PDT, an FCE was observed in the subfoveal region along with a shrinkage of the tumor (Figures 3C, 3D). The patient complained of a worsening of his metamorphopsia with a horizontal distortion of 1.2° and vertical distortion of 1.2°, as measured on the M-chart. But his BCVA was stable at 1.0. Ten months after the onset of FCE, his BCVA remained at 1.0 and his metamorphopsia did not worsen.

## Discussion

We reported a case of an extrafoveal CO that was treated with PDT which resulted in partial decalcification. However, the size of the tumor increased over the next five years and an FCE was formed which included the central fovea as the tumor decalcified.

Earlier studies have reported that the visual prognosis is adversely impacted when the CO is extended over the central fovea. Thus, following the location of the tumor is a critical factor for long-term visual outcomes [7]. Despite the initial extrafoveal location, COs can enlarge in 22% of the cases within five years and in 51% of the cases within 10 years. Conversely, spontaneous decalcification can occur which leads to tumor regression in 28% of cases at five years and in 46% of cases at 20 years [7]. Interestingly, none of the 15 patients with partially decalcified COs at the initial diagnosis had an enlargement of the tumor during the follow-up period. This indicated that decalcification may stabilize the tumor site [7]. Consequently, therapies promoting decalcification have been explored, and PDT has emerged as a promising option [11,13]. In a cohort of nine patients who received PDT, decalcification was observed in all with an average of 73% of the irradiated area decalcified with a significant regression when compared to the natural progression of a CO [11]. This underscores the possibility that PDT may prevent an extrafoveal CO from expanding into the central fovea. On the other hand, tumor growth occurred in 6 to 38 months after PDT in three cases with partial regression, although the site of expansion was not reported [11]. In our case, PDT was performed on the entire CO away from the central fovea, and some decalcification occurred, but the tumor had enlarged 12 months after the full-dose PDT. From the three previous cases and our case, there is a difference in the degree of decalcification between PDT-induced decalcification and spontaneous decalcification which may be related to the course of the CO.

The exact mechanism by which PDT causes a decalcification of COs has not been determined. However, it is known that PDT causes a release of free radicals when the photosensitizer is activated by laser energy. The free radicals damage endothelial cells through an upregulation of immunomodulatory factors, vasoconstriction, and the induction of thrombosis. PDT can affect choroidal vessels and cause choroidal thinning and choroidal and RPE atrophy. RPE atrophy is particularly likely to occur in age-related macular degeneration patients with thin choroids [14]. Although the absence of RPE changes on the CO has been reported as a significant clinical finding predicting CO growth [7], the choroid is thicker at the site of the CO lesion due to the calcification and the vascular structure of the CO [15]. Therefore, PDT caused less damage to the RPE in our case, and an expansion could have also occurred in the CO after PDT.

Decalcification impairs choroidal perfusion and RPE function, and PDT-induced decalcification in subfoveal lesions is detrimental to visual prognosis [13]. In the 12 eyes that had no decalcification at the initial examination but had decalcification during the disease process, there was an expansion in the direction of no decalcification and no tumor expansion from the decalcified area [7]. In our case, CO expansion originated from the partially undecalcified margins near the fovea, which was a partially not decalcified area similar to that reported [7].

FCE is a focal choroidal depression that can occur in diseases such as central serous chorioretinopathy and polypoidal choroidal vasculopathy [16]. Still, in CO, it is caused by tissue loss associated with decalcification of the tumor and displacement of the outer retina and choroid outward [9]. In our case, the FCE was formed by decalcification of the CO, including the central foveal area. The hemorrhage and exudation caused by CNV did not extend into the central fovea, suggesting that the damage to the RPE and outer retinal layer was mild and the visual acuity was preserved. However, because of the short time since tumor regression, there is a possibility that the RPE and photoreceptor cells may degenerate in the future. This would then affect visual acuity, necessitating careful follow-up.

## Conclusions

This clinical case highlights that a CO that partially decalcifies after PDT can still expand and decalcify to form an FCE over several years. Currently, there are no reported methods to control CO expansion other than to promote decalcification by PDT. However, there is no effective strategy to reliably control the expansion into the central fovea over a long period, as this case demonstrates. Therefore, CO requires careful and long-term follow-up examinations, and multiple rounds of PDT may be necessary during the disease. Moreover, improving treatment options other than PDT is needed.
